# Trichinella spiralis

**DOI:** 10.3201/eid2712.211230

**Published:** 2021-12

**Authors:** Monika Mahajan

**Affiliations:** Postgraduate Institute of Medical Education and Research, Chandigarh, India

**Keywords:** Trichinella spiralis, nematode, roundworm, parasite, trichinellosis, James Paget, Richard Owen, Alcide Raillet, Rudolf Virchow, sandy diaphragm, collagen capsule

## *Trichinella spiralis *[tri·kuh·neh′·luh spr·a′·luhs]

**Figure 1:**
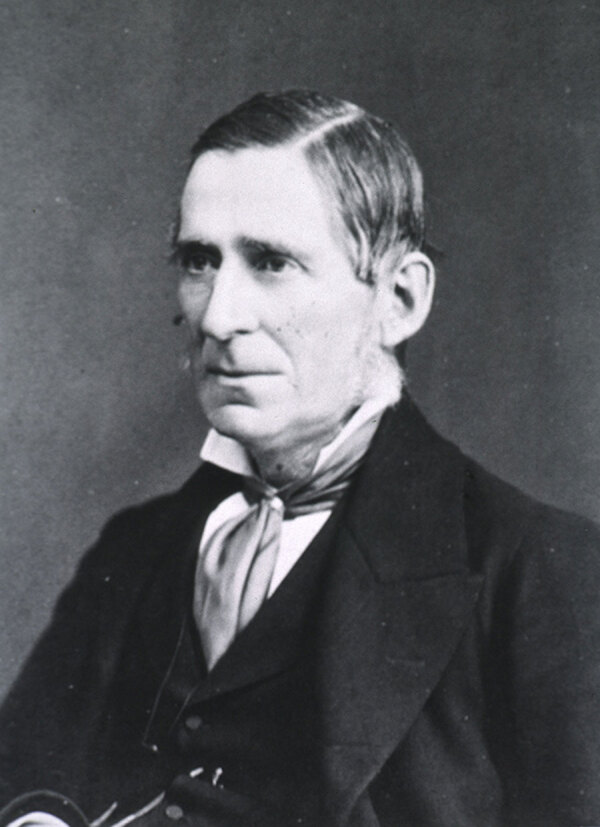
Sir James Paget (January 11, 1814–December 30, 1899), English surgeon and pathologist who observed a spiral encysted nematode in a cadaver. Source: https://resource.nlm.nih.gov/101425853.

**Figure 2:**
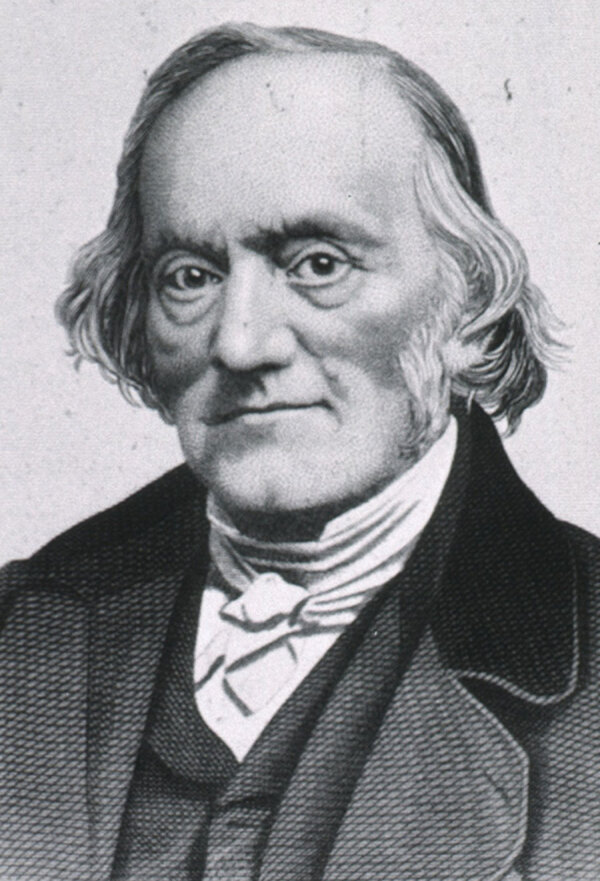
Sir Richard Owen (July 20, 1804–December 18, 1892), English biologist, comparative anatomist, and paleontologist who did not share the credit of discovery of *Trichina spiralis* with Paget. Source: https://resource.nlm.nih.gov/101424684.

**Figure 3:**
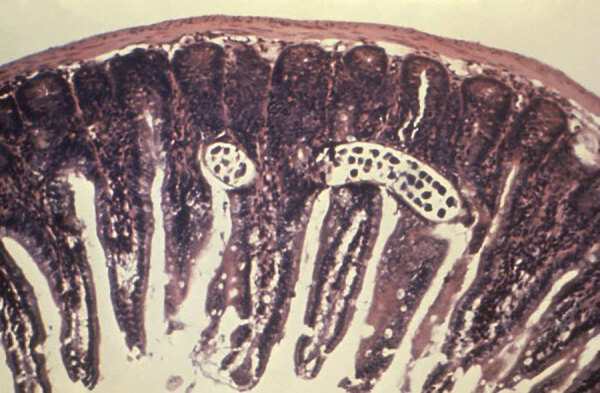
Photomicrograph of an intestinal mucosa tissue specimen showing a *Trichinella spiralis* parasitic nematode, which had burrowed itself into the columnar epithelial intestinal lining, in a case of trichinosis. Source: CDC/Dr. Robert Kaiser (https://phil.cdc.gov/Details.aspx?pid=14931).

*Trichinella* is derived from the Greek words *trichos* (hair) and *ella* (diminutive); *spiralis* means spiral. In 1835, Richard Owen (1804–1892) (Figure 1) and James Paget (1814–1899) (Figure 2) described a spiral worm (*Trichina spiralis*)‒lined sandy diaphragm of a cadaver. In 1895, Alcide Raillet (1852–1930) renamed it as Trichinella spiralis because *Trichina* was attributed to an insect in 1830. In 1859, Rudolf Virchow (1821–1902) described the life cycle. The genus includes many distinct species, several genotypes, and encapsulated and nonencapsulated clades based on the presence/absence of a collagen capsule (Figure 3).

**Figure 4:**
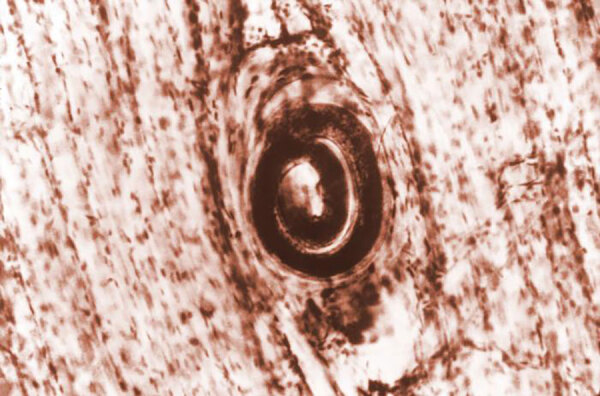
Photomicrograph showing a *Trichinella spiralis* cyst that was embedded in a muscle tissue specimen, in a case of trichinellosis acquired by ingesting meat containing cysts (encysted larvae) of *Trichinella* sp. Source: CDC/Dr. Irving Kagan (https://phil.cdc.gov/Details.aspx?pid=10180).

The smallest, viviparous nematode or pig parasite has sylvatic and domestic cycles and causes trichinellosis or trichinosis. Transmission occurs through the consumption of meat infected with pathogenic cysts, encasing larvae (Figure 4). Human-to-human transmission has not been reported.
